# Supplementary material for: Panproteome-wide analysis of antibody responses to whole cell pneumococcal vaccination
Source: eLife. 2018 Dec 28;7:e37015. doi: 10.7554/eLife.37015 (PMC6344088; doi:10.7554/eLife.37015)
Supplement: Supplementary file 3. — This multivariable logistic binary regression analysis fitted a model combining the explanatory variables of different protein characteristics to the binary dependent variable of whether or not a protein provoked an elevated IgG response, based on the probes listed in Supplementary file 2. The analysis removed variables preventing a maximum likelihood estimate, and the fitted model was refined by stepwise model selection based on Akaike information criterion (AIC) values. The table lists the features found to significantly associate with being identified as inducing a WCV-induced response: the protein’s length, having a signal peptide for secretion, and possessing the listed functional motifs. The lipoprotein motif and SNP_bac_3 domains are associated with the solute-binding proteins of transporters, and the Transpeptidase domain is associated with cell wall metabolism proteins. [file elife-37015-supp3.docx]

| **Variable** | **Estimate** | **Standard Error** | **Z Value** | **Pr(>\|z\|)** |
| --- | --- | --- | --- | --- |
| (Intercept) | -4.09467 | 0.22944 | -17.8460 | < 2x10^-16^ |
| Length | 0.00045 | 0.00015 | 3.07400 | 0.00211 |
| Signal peptide | 1.91052 | 0.43954 | 4.34700 | 1.38x10^-5^ |
| Lipoprotein motif | 0.98702 | 0.54215 | 1.82100 | 0.06867 |
| GntR | 2.36508 | 1.13245 | 2.08800 | 0.03676 |
| SBP_bac_3 | 1.74642 | 0.81619 | 2.14000 | 0.03238 |
| Transpeptidase | 4.48433 | 1.14173 | 3.92800 | 8.58x10^-5^ |
