# Supplementary material for: Panproteome-wide analysis of antibody responses to whole cell pneumococcal vaccination
Source: eLife. 2018 Dec 28;7:e37015. doi: 10.7554/eLife.37015 (PMC6344088; doi:10.7554/eLife.37015)
Supplement: Transparent reporting form [file elife-37015-transrepform.docx]

***eLife’s* transparent reporting form**

We encourage authors to provide detailed information *within their submission* to facilitate the interpretation and replication of experiments. Authors can upload supporting documentation to indicate the use of appropriate reporting guidelines for health-related research (see [EQUATOR Network](http://www.equator-network.org/%20)), life science research (see the [BioSharing Information Resource](https://biosharing.org/)), or the [ARRIVE guidelines](http://www.plosbiology.org/article/info:doi/10.1371/journal.pbio.1000412) for reporting work involving animal research. Where applicable, authors should refer to any relevant reporting standards documents in this form.

If you have any questions, please consult our Journal Policies and/or contact us: [editorial@elifesciences.org](mailto:editorial@elifesciences.org).

**Sample-size estimation**

- You should state whether an appropriate sample size was computed when the study was being designed
- You should state the statistical method of sample size computation and any required assumptions
- If no explicit power analysis was used, you should describe how you decided what sample (replicate) size (number) to use

Please outline where this information can be found within the submission (e.g., sections or figure legends), or explain why this information doesn’t apply to your submission:

This study used all available serum samples from the phase I clinical trial, which was intended to assess the safety and immunogenicity of the *Streptococcus pneumoniae* RM200 whole cell vaccine. The trial was intended to provide preliminary data for subsequent larger trials, and a power calculation was not made during the trial design, nor in the selection of samples for this study. This is stated in the Methods section: “*This study is a post hoc analysis of the samples available at the end of the clinical trial, and does not present the original safety and immunogenicity tests for which the trial was designed. No specific power analysis was conducted prior to the trial, or the sample selection for this subsequent study.*”.

**Replicates**

- You should report how often each experiment was performed
- You should include a definition of biological versus technical replication
- The data obtained should be provided and sufficient information should be provided to indicate the number of independent biological and/or technical replicates
- If you encountered any outliers, you should describe how these were handled
- Criteria for exclusion/inclusion of data should be clearly stated
- High-throughput sequence data should be uploaded before submission, with a private link for reviewers provided (these are available from both GEO and ArrayExpress)

Please outline where this information can be found within the submission (e.g., sections or figure legends), or explain why this information doesn’t apply to your submission:

Each available timepoint from each individual was treated as an independent biological replicate. These samples are described in Figure 1.

Only a single technical replicate was available for each biological replicate. Comparisons between the results for samples analysed with a pilot version of the array, and the version used in this study, found a stronger correlation of IgG binding measurements between technical replicates than when comparing other samples (Figure 2-figure supplement 1). This higher correlation also applied to samples from the same individual at different timepoints, which explains the ability to detect antibody ‘fingerprints’ in Figure 2. Therefore technical replicates were not included in the main analyses presented in this work, in order to maximise the number of biological replicates, given the funding available.

We have now added this information to the Results: “*No technical replicates were included, as analysis of pilot data obtained using a smaller version of the array found reproducible differences between individuals (Figure 2-figure supplement 1). This suggested results were consistent between array assays of samples from the same individual, and that the study should span the maximal number of trial participants, as between-individual variation might be an important factor in understanding the response to the WCV.*”.

Of the 304,590 probe signals included in this study, 152 normalized to a value below -2 (less than 0.25 of the *in vitro* transcription and translation control probe signals), and were adjusted to -2. A further five measurements were missing, and were omitted from the analyses. This information is now provided in the Methods: “*Values below -2 in the normalised data, corresponding to less than 0.25 of the IVTT control probe signals, were adjusted to -2. This affected 152 of the 304,590 binding values included in the study, of which a further five were missing.*”.

**Statistical reporting**

- Statistical analysis methods should be described and justified
- Raw data should be presented in figures whenever informative to do so (typically when N per group is less than 10)
- For each experiment, you should identify the statistical tests used, exact values of N, definitions of center, methods of multiple test correction, and dispersion and precision measures (e.g., mean, median, SD, SEM, confidence intervals; and, for the major substantive results, a measure of effect size (e.g., Pearson's r, Cohen's d)
- Report exact p-values wherever possible alongside the summary statistics and 95% confidence intervals. These should be reported for all key questions and not only when the p-value is less than 0.05.

Please outline where this information can be found within the submission (e.g., sections or figure legends), or explain why this information doesn’t apply to your submission:

A table of statistical tests in the manuscript is provided below; all this information is included in the manuscript.

(For large datasets, or papers with a very large number of statistical tests, you may upload a single table file with tests, Ns, etc., with reference to sections in the manuscript.)

| **Test and purpose** | **Section and context** | ***N*** | **Test statistic** | ***p* value** |
| --- | --- | --- | --- | --- |
| **Kruskal-Wallis test** - non-parametric test of whether multiple sets of values appear to have the same distribution | **Stable antibody “fingerprints” of vaccinated individuals** and **Figure 4-figure supplement 1** – testing for heterogeneity between cohorts in individuals’ median day 0 IgG binding to the panproteome array | 29 | χ^2^ = 0.20 | 0.98 |
|  | **Stable antibody “fingerprints” of vaccinated individuals** and **Figure 4-figure supplement 1** - testing for heterogeneity between cohorts in individuals’ median Δ_0→84_ | 29 | χ^2^ = 3.17 | 0.37 |
|  | **Stable antibody “fingerprints” of vaccinated individuals** and **Figure 4-figure supplement 1** - testing for heterogeneity between cohorts in individuals’ median Δ_0→84_ to antibody binding proteins | 29 | χ^2^ = 4.63 | 0.20 |
| **Wilcoxon-rank sum test** - non-parametric test of whether two sets of values appear to have the same distribution | **Figure 4-figure supplement 1** - testing for heterogeneity between the placebo group and cohort 3 in individuals’ median day 0 IgG binding to the panproteome array | 16 | *W* = 34 | 0.88 |
|  | **Figure 4-figure supplement 1** - testing for heterogeneity between the placebo group and cohort 3 in individuals’ median Δ_0→84_ | 16 | *W* = 20 | 0.23 |
|  | **Figure 4-figure supplement 1** - testing for heterogeneity between the placebo group and cohort 3 in individuals’ median Δ_0→84_ to antibody binding proteins | 16 | *W* = 14 | 0.065 |
|  | **Stronger WCV responses were associated with ABTs**and **Figure 4A** – testing for differences in Δ_0→84_ between probes from proteins conserved in the WCV, and those absent from it | 2123 | *W* = 403523 | 0.33 |
|  | **Stronger WCV responses were associated with ABTs**and **Figure 4A** – testing for differences in Δ_0→84_ between probes from antibody binding proteins conserved in the WCV, and those absent from it | 127 | *W* = 970 | 0.000065 |
|  | **Figure 4A** – testing for differences in Δ_0→84_ between probes antibody binding proteins conserved in other streptococci, and those not conserved in other streptococci | 87 | *W* = 676 | 0.073 |
|  | **Figure 4-figure supplement 3** – testing for differences in day 0 IgG binding between probes from proteins highly similar in the array and the WCV, and those divergent in, or absent from, the WCV | 2123 | *W* = 358929 | 0.005 |
|  | **Figure 4-figure supplement 3** – testing for differences in day 0 IgG binding between probes from proteins highly similar in the array and the WCV, and those divergent in, or absent from, the WCV | 127 | *W* = 1501 | 0.22 |
|  | **Figure 4-figure supplement 3** – testing for differences in day 0 IgG binding between probes antibody binding proteins conserved in other streptococci, and those not conserved in other streptococci | 87 | *W* = 937 | 0.62 |
|  | **Differing kinetics of WCV responses between antigens** – testing for differences in the pre-WCV IgG binding of probes eliciting a substantial response between days 0 and 28, and between days 56 and 84. | 139 | *W* = 2116.5 | 0.0013 |
|  | **Figure 2-figure supplement 1**  - testing for differences in the distribution of correlation statistics between technical replicates and other samples from the same trial participant | 312 | *W* = 13239 (all probes)  *W* = 13480 (immunoreactive probes) | 0.073 (all probes)  0.036 (immunoreactive probes) |
|  | **Figure 2-figure supplement 1**  - testing for differences in the distribution of correlation statistics between technical replicates and samples from different participants in the same cohort, taken at the same stage of the trial | 601 | *W* = 48170 (all probes)  *W* = 48738 (immunoreactive probes) | <10^-16^ (all probes)  <10^-16^ (immunoreactive probes) |
| **Fisher’s exact test** – test of dependency between categorical variables | **Stronger WCV responses were associated with ABTs** - enrichment of antibody binding proteins in probes associated with Δ_0→84_ above the 0.2 threshold in cohort 1 | 2343 | 5.95-fold enrichment | 6.11x10^-9^ |
|  | **Stronger WCV responses were associated with ABTs** - enrichment of antibody binding proteins in probes associated with Δ_0→84_ above the 0.2 threshold in cohort 2 | 2343 | 4.06-fold enrichment | 2.37x10^-5^ |
|  | **WCV elicits elevated IgG to a specific minority of proteins** - enrichment of antibody binding proteins in probes associated with Δ_0→84_ above the 0.2 threshold in cohort 3 | 2343 | 5.40-fold enrichment | <10^-16^ |
|  | **Consistent identification of antigens by complementary statistical analyses** – testing enrichment of antibody-binding proteins among WCV antigens relative to other immunoreactive proteins | 1443 | 6.16-fold enrichment | <10^-16^ |
|  | **Consistent identification of antigens by complementary statistical analyses** – testing enrichment of antibody-binding proteins among WCV antigens relative to other immunoreactive proteins, among proteins exhibiting high similarity between RM200 and the array | 1062 | 16.01-fold enrichment | <10^-16^ |
|  | **Diverse functional characteristics of proteins eliciting IgG responses** – testing for enrichment of solute-binding proteins among WCV antigens relative to other immunoreactive proteins | 1062 | 7.60-fold enrichment | 1.16x10^-7^ |
|  | **Diverse functional characteristics of proteins eliciting IgG responses** – testing for enrichment of solute-binding proteins among WCV antigens within the set of antibody-binding proteins | 208 | 3.54-fold enrichment | 0.014 |
|  | **Diverse functional characteristics of proteins eliciting IgG responses** – testing for enrichment of cell wall machinery proteins among WCV antigens within the set of antibody-binding proteins | 208 | 3.43-fold enrichment | 0.0014 |
| **Generalised linear model** - used for multivariable regression analysis to identify associations between dependent variables and an outcome | **Diverse functional characteristics of proteins eliciting IgG responses** and **Supplementary file 3** – testing for protein characteristics that distinguish WCV antigens from other proteins exhibiting high similarity between RM200 and the array | 1600 | *Z* = 4.35 | 1.38x10^-5^ |
| **Pearson correlation** – measure of the linear correlation between two sets of variables | **Figure 3** – testing for a correlation between the day 0 IgG binding and Δ_0→84_ in each cohort | 2343 | *R*^2^ = 0.015 (placebo)  *R*^2^ = 0.083  (cohort 1)  *R*^2^ = 0.062  (cohort 2)  *R*^2^ = 0.11 (cohort 3) | 1.7x10^-9^ (placebo)  <10^-16^ (cohort 1)  <10^-16^ (cohort 2)  <10^-16^ (cohort 3) |
|  | **Figure 3** – testing for a correlation betweenΔ_0→84_ for cohort 3 and the other vaccinated cohorts | 2343 | *R*^2^ = 0.48 (cohort 1)  *R*^2^ = 0.33 (cohort 2) | <10^-16^ (cohort 1)  <10^-16^ (cohort 2) |
| **Empirical Bayes** - calculates probabilities relative to a prior distribution estimated from the dataset itself | **Identification of specific antigens** – testing for immunoreactive probes associated with a significantly high Δ_0→84_ from pairwise contrasts of vaccinated cohorts relative to the placebo cohort | 1584 | Values for probes associated with a significant change provided in Supplementary File 2 | |
|  | **Identification of specific antigens** – testing for immunoreactive probes, excluding the diverse core loci, associated with a significantly high Δ_0→84_ from pairwise contrasts of vaccinated cohorts relative to the placebo cohort | 1384 |  |  |
| **Linear mixed effects model** – models outcomes as a noisy linear response to random and fixed effects | **Stable antibody “fingerprints” of vaccinated individuals** – testing for differences in Δ_0→84_ between cohorts at the per probe level, through using ANOVA to compare fits of linear mixed effects models across all 2,343 probes with individual as a random effect, and either probe, or both probe and cohort, as fixed effects. | 2343 | χ^2^ = 8.96 | 0.030 |
|  | **Stable antibody “fingerprints” of vaccinated individuals** – testing for differences in Δ_0→84_ between cohorts at the per probe level, through using ANOVA to compare fits of linear mixed effects models across the 1,584 immunoreactive probes with individual as a random effect, and either probe, or both probe and cohort, as fixed effects. | 1584 | χ^2^ = 11.8 | 0.0081 |
|  | **Identification of specific antigens** – testing for immunoreactive probes associated with a significantly improved model fit when the fixed effects of dose amount, or number of doses, were included in the linear model | 1584 | Values for probes associated with a significant change provided in Supplementary File 2 | |
|  | **Identification of specific antigens** – testing for immunoreactive probes, excluding the diverse core loci, associated with a significantly improved model fit when the fixed effects of dose amount, or number of doses, were included in the linear model | 1384 |  |  |

**Group allocation**

- Indicate how samples were allocated into experimental groups (in the case of clinical studies, please specify allocation to treatment method); if randomization was used, please also state if restricted randomization was applied
- Indicate if masking was used during group allocation, data collection and/or data analysis

The analysed samples originated in a double-blind randomised placebo-controlled trial. In the Methods section, we describe the randomisation of participants in the clinical trial: “*Forty-two participants were enrolled and either received a WCV dose or a saline placebo, with sequential subject assignment performed by data management using an electronic randomization block design. Pharmacy staff responsible for preparing and administering vaccinations were unblinded. All others involved in conducting the trial, including participants, remained blinded to treatment assignment.”* Statistical analysis of the array data was unblinded.

Please outline where this information can be found within the submission (e.g., sections or figure legends), or explain why this information doesn’t apply to your submission:

**Additional data files (“source data”)**

- We encourage you to upload relevant additional data files, such as numerical data that are represented as a graph in a figure, or as a summary table
- Where provided, these should be in the most useful format, and they can be uploaded as “Source data” files linked to a main figure or table
- Include model definition files including the full list of parameters used
- Include code used for data analysis (e.g., R, MatLab)
- Avoid stating that data files are “available upon request”

For Figure 1-figure supplement 1, Figure 4-figure supplement 4, Figure 4-figure supplement 5, Figure 4-figure supplement 10 and Figure 4-figure supplement 13, analyses were performed on previously published, publically available sequence data, in conjunction with the RM200 genome assembly, which has been uploaded to the European Nucleotide Archive with accession code ERS2169631. All other figures were generated using the proteome array data, which has now been uploaded as two source data files, as specified in the Methods section: “*Information on the samples has been provided as Figure 1-source data 1, and the standardized IgG binding values from the proteome array have been provided as Figure 2-source data 1.*”.

Please indicate the figures or tables for which source data files have been provided:
